# A Cross-Sectional Study to Evaluate the Effectiveness of Patient Information Guides Produced by ChatGPT Versus Google Gemini for Three Pediatric Illnesses

**DOI:** 10.7759/cureus.88824

**Published:** 2025-07-26

**Authors:** Kavya Thayappa, Renee Manake, Sai Brigisha Kyasa, Aishwarya Babu

**Affiliations:** 1 Pediatrics, MVJ Medical College and Research Hospital, Bengaluru, IND; 2 Internal Medicine, Makerere University College of Health Sciences, School of Medicine, Kampala, UGA; 3 Pediatrics, Kamineni Institute of Medical Sciences, Nalgonda, IND; 4 Internal Medicine, Tbilisi State Medical University, Tbilisi, GEO

**Keywords:** artificial intelligence, chatgpt, educational tool, google gemini, patient education brochure, pediatric diseases

## Abstract

Objectives: Educating pediatric patients and their caregivers about the disease is crucial for improving treatment adherence, recognizing complications early, and alleviating anxiety. AI tools such as ChatGPT and Google Gemini offer personalized education, benefiting patients and providers, and are increasingly utilized in healthcare. This study aims to compare patient education guides created by ChatGPT and Google Gemini for acute otitis media, pneumonia, and pharyngitis.

Methods: Patient information guides on pediatric diseases generated by ChatGPT and Google Gemini were evaluated by comparing various variables (words, sentences, average words per sentence, average syllables per word, grade level, and ease score) and further assessed for ease using the Flesch-Kincaid calculator, similarity using Quillbot, and reliability using the Modified Discern score. Statistical analysis was done using R v4.3.2.

Results: Both tools' responses were statistically compared. No significant difference was found in word count (ChatGPT: 477.3; Google Gemini: 394.0; p=0.0765) or sentences (ChatGPT: 35.33; Google Gemini: 46.33; p=0.184). Google Gemini scored slightly higher in ease (ChatGPT: 37.79; Google Gemini: 57.10) and grade level (ChatGPT: 11.40; Google Gemini: 7.43), but these were not statistically significant (p>0.05), indicating no clear superiority.

Conclusions for practice: In a comparison of patient education guides created by both tools for acute otitis media, pneumonia, and pharyngitis, there was no statistically significant difference to determine the superiority of one AI tool over the other. Further studies should comprehensively evaluate various AI tools across a broader range of diseases. It is also important to assess whether AI tools can provide real-time, verifiable content based on the latest medical advancements.

## Introduction

Pediatric illnesses can be particularly worrying, especially for first-time parents who are navigating the complexities of childcare for the first time. In today's digital age, parents increasingly rely on digital sources of information, particularly with the advent of artificial intelligence (AI) [1]. AI has the computational power to assess new information based on previously analyzed data in real-time. The benefits of integrating AI tools in medicine include aiding in the evaluation of radiological images, pathology slides, and electronic medical records, thereby speeding up diagnosis and treatment processes [2].

Among the AI tools available, ChatGPT and Google Gemini are prominent large language models with distinct functionalities. ChatGPT, utilizing GPT-3 and GPT-4 models since 2022, is primarily pretrained on text and code, excelling in generating creative outputs such as poems and scripts, though it cannot edit responses once sent [3,4]. Conversely, Google Gemini, part of Google’s LaMDA (Language Model for Dialogue Applications), family launched in 2023, is pretrained on text, codes, image generation, and real-time web access, offering concise information and functioning as a research assistant with the capability to edit responses post-submission [4,5].

This study explores three common pediatric illnesses: acute otitis media, acute pharyngitis, and pneumonia. Acute otitis media presents with otalgia, fever, erythema of the tympanic membrane, and middle ear effusion, affecting 80% of children before age three [6]. Acute pharyngitis involves the inflammation of the posterior pharynx and tonsils, caused by various viruses and bacteria [7]. Pneumonia, an infection of the lung parenchyma, is the most common cause of death after 28 days of birth [8,9].

Given the prevalence of these conditions, patient education guides play a crucial role in preventing complications, such as using spirometry in children with neutropenia to prevent pneumonia [10]. AI tools have helped individuals in a better understanding of the common pediatric conditions and to take part in shared decision-making [11]. The growing use of AI tools in empowering the medical field provoked us to conduct a study on AI tools as a platform for patient education.

Aims and objectives

To compare the effectiveness of ChatGPT and Google Gemini in generating patient education guides for three common pediatric illnesses, acute otitis media, pharyngitis, and pneumonia, by evaluating ease of understanding and readability.

## Materials and methods

A cross-sectional original research study was conducted over a period of one week from 24th March, 2024, to 30th March, 2024. The study was deemed exempt from ethics committee approval, in view of no human participant data.

Data collection and evaluation

First, the responses were generated from ChatGPT 3.5 and Google Gemini. We selected three common pediatric illnesses: acute otitis media, pharyngitis, and pneumonia. The following prompts were given to the AI tools: “Write a patient education guide for Acute Otitis Media/Pharyngitis/Pneumonia,” respectively. All responses generated were collected in a Microsoft Word (Microsoft, Redmond, WA) document (Tables 2-4 of Appendices).

The responses generated were then graded using various tools. The Flesch-Kincaid calculator was used for assessing the word count, sentence count, ease of understanding, and readability of the information generated [12]. The Quillbot plagiarism checker was used to assess the similarity to content already published on the web, in books, or research papers [13]. Finally, the Modified DISCERN score was used to check the reliability of the scientific text. The DISCERN score is a validated tool that was designed to assess the quality of written health information, evaluating factors such as reliability, accuracy, and comprehensiveness [14].

The data was then exported to Microsoft Excel (Microsoft, Redmond, WA), and statistical analysis was done using R version 4.3.2. The responses generated by ChatGPT and Google Gemini were compared using the Wilcox rank sum test, with p-value <0.05 considered significant. The correlation between ease score and reliability score was further compared using Pearson’s coefficient of correlation.

## Results

ChatGPT and Google Gemini were used to generate brochures on patient education for three pediatric diseases: acute otitis media, pharyngitis, and pneumonia.

Table 1 shows characteristics of responses generated by both ChatGPT and Google Gemini for the dataset considered. The characteristics considered were the word count, sentence count, average words per sentence, average syllables per word, grade level, ease score, similarity percentage, and reliability score. The mean ease scores and grade levels of responses generated by Google Gemini were better compared to those generated by ChatGPT. The Wilcoxon rank sum test (non-parametric) was used to compare the medians between ChatGPT and Google Gemini, due to the data not being normally distributed. There was no significant difference observed in the word count (p=0.0765), sentence count (p=0.184), average word per sentence (p=0.0765), average syllables per word (p=0.3687), similarity percentage (p=0.7), and reliability score (p=0.6193).

**Table 1:** Characteristics of responses generated by ChatGPT and Google Gemini

Variables	ChatGPT		Google Gemini		P-value*
	Mean	Standard Deviation	Mean	Standard Deviation	
Words	477.3	4.61	394.0	23.64	0.0765
Sentences	35.33	5.77	46.33	7.51	0.184
Average words per sentence	13.73	2.19	8.60	0.85	0.0765
Average syllables per word	1.83	0.12	1.67	0.23	0.3687
Grade level	11.40	2.25	7.43	2.46	0.184
Ease score	37.79	11.96	57.10	18.80	0.4
Similarity %	52.57	29.56	41.7	31.11	0.7
Reliability Score	2.67	0.58	2.33	0.58	0.6193
*Wilcoxon’s rank sum test. P-values <0.05 are considered statistically significant.					

**Figure 1:**
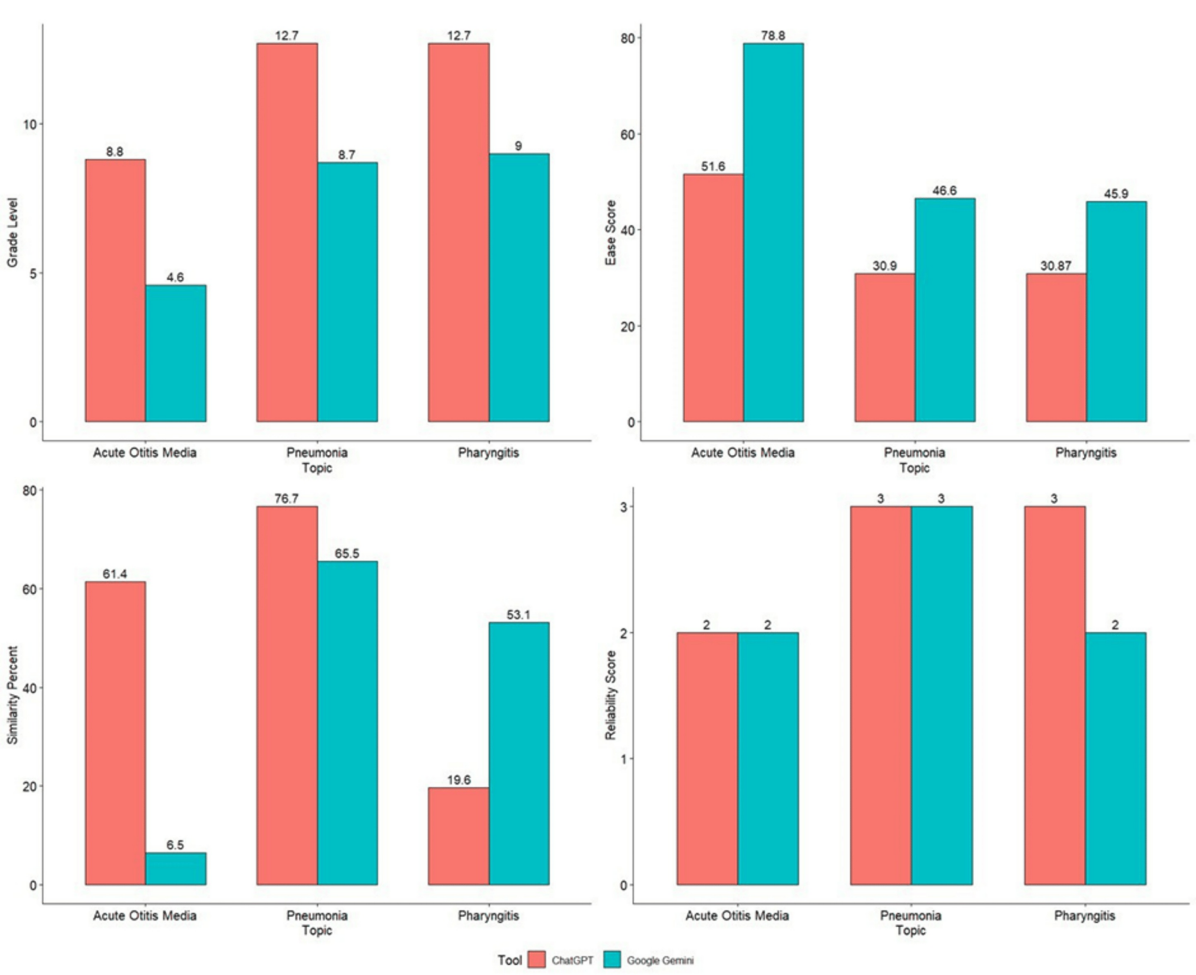
Graphical representation comparing grade level, ease score, similarity percentage, and reliability score for patient education guides generated by ChatGPT and Google Gemini

Figure 1 shows a graphical representation of the mean value comparison for grade level, ease score, similarity percentage, and reliability score in the brochures generated by ChatGPT and Google Gemini for the three pediatric diseases. In the plots, ChatGPT results are depicted in red, while Google Gemini results are shown in blue. The horizontal axis represents the three pediatric diseases considered, and the vertical axis corresponds to the characteristic being visualized. ChatGPT shows higher grade-level values across all three diseases. Conversely, Google Gemini demonstrates higher ease scores than ChatGPT for all three diseases. Typically, a higher ease score correlates with a lower grade level, a trend supported by the figure. The reliability scores remain consistent between ChatGPT and Google Gemini for all three diseases, while the similarity percentages vary between the two AI tools across the diseases.

## Discussion

This is a cross-sectional study comparing responses from ChatGPT and Google Gemini for pediatric patient education brochures on acute otitis media, pneumonia, and pharyngitis. It was found that there was no statistically significant difference in key parameters. Both tools yielded comparable results in average syllables per count and reliability score. However, ChatGPT exhibited slightly higher metrics in words, average words per sentence, grade level, and similarity, while Google Gemini scored slightly higher in sentences and ease score.

Tools such as ChatGPT and Google Gemini generate text-based content using natural language, offering significant potential for patient education within healthcare contexts [15]. Patient education brochures should be crafted at a readability level below that commonly attained in high school, ensuring wide accessibility. Using non-complex language, shorter texts with simpler, concise language are more effective in improving pediatric patient education. This strategy aims to enable most parents to understand the content, thus supporting the creation of easily comprehensible brochures [16,17]. 

Our study revealed that Google Gemini generated patient education material with a mean ease score of 57.10, corresponding to a mean grade level of 7.43. In contrast, ChatGPT generated patient education material with a mean ease score of 37.79 and a mean grade level of 11.40. Based on the scores, a high school student would be able to interpret the Gemini data, while it would be difficult for a high school student to interpret ChatGPT data. While these differences suggest better performance by Gemini compared to ChatGPT, it is important to note that our analysis is based on only three samples, and the data is not normally distributed. As such, this information does not provide sufficient evidence to conclude the superiority of one AI tool over the other. A study on patient education for dermatological conditions using ChatGPT found that it generates text suitable for high school to college-level readers but exhibits higher-than-acceptable text similarity, warranting caution. Authors should use ChatGPT judiciously, ensuring thorough plagiarism checks [18].

The data generated by AI tools like ChatGPT and Google Gemini may pose legal and ethical concerns, as they are trained on existing literature, potentially leading to text similarity and plagiarism [19,20]. Plagiarism is detrimental in medical science as it reflects misconduct and a breach in the scientific research and its integrity [20]. In our study, the mean value for similarity percent for the responses generated by ChatGPT was 52.57% and for Google Gemini was 41.7%. Additionally, the sourcing of information from unreliable platforms such as non-healthcare blogs raises doubts about data integrity, further compounded by the lack of transparency surrounding AI-generated responses; therefore, vigilant oversight must be ensured.

The modified DISCERN score, a reliable tool for evaluating health care information, was used in our study to assess the online/media content for patient education materials. This subjective scoring method entails researchers assigning points based on yes or no responses to a set of five questions, with each “yes” awarded one point and each “no” scoring zero. A higher total score indicates greater reliability [20-22]. In our study, the mean reliability score was 2.68 and 2.33 for ChatGPT and Google Gemini, respectively, indicating no significant differences in the average DISCERN score between the content generated by the AI tools used. Therefore, further research is needed to validate the reliability of AI-generated content.

Limitations

In this study, only two AI tool models were evaluated, thus limiting our findings. Additionally, our study focused on a limited number of pediatric diseases, requiring further investigation across a broader range of medical conditions. Furthermore, the latest versions of the AI tools were not used, which may impact the relevance and accuracy of the content generated. Therefore, further studies are warranted to determine the suitability of AI tools in generating patient education information.

## Conclusions

A study on the suitability of using AI tools for generating patient education brochures for common pediatric diseases was conducted. ChatGPT and Google Gemini were the AI tools considered for generating texts of the three most common pediatric diseases. This study highlights that there are no significant differences in words, sentences, the average syllables per count, and the reliability score of responses generated by the two AI tools for patient information brochures for acute otitis media, pharyngitis, and pneumonia in the pediatric age group. The superiority of one tool over the other is inconclusive from the study.

Additional studies must be carried out to gain more insights from the AI tools for broader diseases. It should also be assessed whether these tools create content using the updated medical guidelines. There is potential for improving the capacity of the AI tools to provide accurate and up-to-date information that has to be verified before being used by the public on a large scale.

## Appendices

**Table 2:** Patient education guides generated for “acute otitis media”

Write a patient education guide for “Acute Otitis Media”		
AI	ChatGPT	Google Gemini
Response	What is Acute Otitis Media? Acute otitis media (AOM) is a common ear infection that affects the middle ear, which is the space behind the eardrum. It often occurs in children and can cause discomfort and pain. Causes: AOM usually develops after a bacterial or viral infection, such as a cold, flu, or respiratory infection. These infections can lead to the inflammation and swelling of the Eustachian tube, which connects the middle ear to the back of the throat. When this tube becomes blocked, fluid can build up in the middle ear, creating a perfect environment for bacteria or viruses to grow, leading to an ear infection. Symptoms: Ear pain or discomfort Tugging or pulling at the ear Fever Difficulty sleeping Irritability or fussiness Fluid draining from the ear Hearing difficulties or temporary hearing loss Loss of appetite Balance problems or dizziness Treatment: Treatment for AOM may vary depending on the severity of symptoms and the child's age. It's essential to consult a healthcare professional for proper diagnosis and treatment. Treatment options may include: Pain Management: Over-the-counter pain relievers, such as acetaminophen or ibuprofen, can help alleviate ear pain and reduce fever. Antibiotics: If the infection is bacterial or if symptoms are severe, your doctor may prescribe antibiotics to help fight the infection. Ear Drops: Prescription or over-the-counter ear drops may be recommended to relieve pain or reduce inflammation. Rest and Fluids: Encourage your child to rest and drink plenty of fluids to help their body fight off the infection. Warm Compress: Applying a warm compress to the affected ear can help relieve pain and discomfort. Prevention: While it's not always possible to prevent AOM, there are some steps you can take to reduce your child's risk: Vaccinations: Ensure your child is up-to-date on their vaccinations, as some vaccines can help prevent infections that may lead to AOM. Hand Hygiene: Encourage regular handwashing to prevent the spread of germs. Avoiding Secondhand Smoke: Exposure to secondhand smoke can increase the risk of ear infections, so it's essential to keep your child away from smoke. Breastfeeding: If possible, breastfeed your baby, as breast milk contains antibodies that can help boost their immune system and reduce the risk of infections. Limiting Pacifier Use: Limit the use of pacifiers, especially once your child reaches six months of age, as prolonged pacifier use can increase the risk of ear infections. When to See a Doctor: It's essential to contact your child's healthcare provider if you suspect they have AOM or if they experience: Severe or prolonged ear pain High fever Fluid draining from the ear Symptoms that worsen or don't improve with treatment Remember, timely diagnosis and treatment are crucial for managing AOM and preventing complications. Your child's healthcare provider can provide personalized recommendations based on their specific needs.	Earaches and Ouchies: A Guide to Acute Otitis Media (AOM) for Kids! What is AOM? AOM, or acute otitis media, is a fancy way of saying your middle ear (the space behind your eardrum) is infected! This infection can make your ear feel yucky and cause some pain. How do I know if I have AOM? Here are some clues: Earache: This is the most common symptom. It might feel like a throbbing or a sharp pain. Fever: You might feel warm or have a temperature above normal. Ear drainage: Sometimes pus or fluid might leak out of your ear. Trouble sleeping: The pain might make it hard to catch those Zzzs. Feeling fussy: You might not feel like yourself and be a little cranky. Not all earaches are AOM! Sometimes, earaches can be caused by other things, like swimming pool water or a scratch in your ear canal. If you're not sure what's causing your earache, it's always best to see your doctor! What will the doctor do? The doctor will take a peek at your ear with a special tool called an otoscope. It looks a little like a flashlight and lets the doctor see if your eardrum is red and swollen. Do I need medicine? Sometimes, your body can fight off the infection on its own. But if your AOM is really bad, your doctor might prescribe antibiotics, which are medicines that kill germs. Feel better tips! Here are some things that can help you feel better while your ear heals: Rest: Get plenty of sleep to help your body fight the infection. Pain relief: Ask your parents about medicine like acetaminophen or ibuprofen to help with the pain (be sure to follow the dosage instructions carefully!). Warm compress: A warm washcloth on your ear can feel soothing. Call your doctor if: Your earache gets worse after a day or two. You have a fever that won't go down. You have pus or fluid draining from your ear for more than a few days. You feel dizzy or have trouble hearing. Remember: Earaches can be no fun, but with a little care and maybe some cuddles from Mom or Dad, you'll be feeling like yourself again in no time!

**Table 3:** Patient education guides generated for “pneumonia”

Write a patient education guide for “Pneumonia”	
Chat GPT	Google Gemini
Title: Understanding Pneumonia: A Patient Education Guide Introduction: Pneumonia is a common respiratory infection that affects the lungs. It can range from mild to severe and can be caused by various infectious agents, including bacteria, viruses, and fungi. Understanding pneumonia, its symptoms, causes, treatment, and prevention is essential for effective management and recovery. This guide aims to provide you with valuable information about pneumonia and empower you to take control of your health. What is Pneumonia? Pneumonia is an infection that inflames the air sacs in one or both lungs. The air sacs may fill with fluid or pus, causing cough with phlegm or pus, fever, chills, and difficulty breathing. Pneumonia can affect people of all ages, but it is most serious for infants, young children, older adults, and people with weakened immune systems. Types of Pneumonia: Community-acquired pneumonia: This type of pneumonia occurs outside of healthcare settings and is typically caused by bacteria or viruses. Hospital-acquired pneumonia: Also known as nosocomial pneumonia, this type occurs during a hospital stay and is often caused by bacteria that are resistant to antibiotics. Aspiration pneumonia: This occurs when you inhale food, drink, saliva, or vomit into your lungs, leading to infection. Viral pneumonia: Caused by viruses such as influenza, respiratory syncytial virus (RSV), or adenovirus. Bacterial pneumonia: Caused by bacteria such as Streptococcus pneumoniae, Mycoplasma pneumoniae, or Haemophilus influenzae. Fungal pneumonia: Caused by fungi such as Pneumocystis jirovecii, particularly in people with weakened immune systems. Symptoms of Pneumonia: The symptoms of pneumonia can vary depending on the cause, severity, and individual factors. Common symptoms include: Cough, often with phlegm or pus Fever Chills Difficulty breathing or shortness of breath Chest pain when breathing or coughing Fatigue Nausea, vomiting, or diarrhea Confusion or changes in mental awareness (in older adults) Diagnosis: If your healthcare provider suspects pneumonia, they may perform a physical examination, listen to your lungs with a stethoscope, and order diagnostic tests such as: Chest X-ray Blood tests, including a complete blood count (CBC) and blood cultures Sputum culture and sensitivity test (to identify the cause of infection) Pulse oximetry (to measure oxygen levels in your blood) CT scan of the chest (in some cases) Treatment: The treatment for pneumonia depends on the type, severity, and underlying cause of the infection. It may include: Antibiotics (for bacterial pneumonia) Antiviral medications (for viral pneumonia) Antifungal medications (for fungal pneumonia) Over-the-counter or prescription cough medicines Fever reducers such as acetaminophen or ibuprofen Oxygen therapy (in severe cases) Intravenous (IV) fluids and nutrition (for hospitalized patients) Rest and plenty of fluids In some cases, hospitalization may be necessary, especially for severe pneumonia or if you have underlying health conditions. Prevention: Preventing pneumonia involves several strategies, including: Getting vaccinated: Vaccines are available to prevent certain types of pneumonia, including the pneumococcal vaccine and the flu vaccine. Practicing good hygiene: Wash your hands frequently with soap and water, avoid close contact with sick individuals, and cover your mouth and nose when coughing or sneezing. Quitting smoking: Smoking damages the lungs and increases the risk of respiratory infections, including pneumonia. Managing underlying health conditions: Properly managing conditions such as asthma, COPD, diabetes, and heart disease can help reduce the risk of pneumonia. Strengthening your immune system: Eat a healthy diet, exercise regularly, get enough sleep, and reduce stress to support your immune system's function. Conclusion: Pneumonia is a serious respiratory infection that requires prompt diagnosis and treatment. By understanding the symptoms, causes, diagnosis, treatment, and prevention strategies, you can take proactive steps to protect yourself and your loved ones from pneumonia. If you experience symptoms of pneumonia, seek medical attention promptly to receive the appropriate care and support for your recovery.	Understanding Pneumonia What is Pneumonia? Pneumonia is an infection that inflames the air sacs in your lungs. These air sacs, called alveoli, fill with fluid or pus (thick, yellow fluid) which makes breathing difficult. Pneumonia can be caused by bacteria, viruses, or even inhaling harmful substances. What are the Symptoms? Common symptoms of pneumonia include: Cough, possibly with phlegm (mucus) that may be green, yellow, or bloody Fever, sweating, and chills Chest pain that worsens when you breathe or cough Shortness of breath Rapid breathing Fatigue and feeling tired Who is at Risk? Anyone can get pneumonia, but some people are at higher risk, including: Infants and young children Adults over 65 People with chronic health conditions like asthma, heart disease, or diabetes Smokers People who have recently been hospitalized Diagnosing Pneumonia Your doctor will ask about your symptoms and medical history. They may also perform a physical exam, chest X-ray, or other tests to diagnose pneumonia and determine the cause. Treatment Options Treatment for pneumonia depends on the severity of your illness and the cause. Most cases of bacterial pneumonia can be treated with antibiotics taken by mouth. For viral pneumonia, rest, fluids, and medications to relieve symptoms are usually recommended. In severe cases, hospitalization may be necessary for oxygen therapy or intravenous antibiotics. Recovery and Prevention Getting plenty of rest, drinking fluids, and taking your medications as prescribed are all important for recovery. Here are some tips to help prevent pneumonia: Wash your hands frequently with soap and water or use an alcohol-based hand sanitizer. Get a flu shot every year and ask your doctor about the pneumococcal vaccine, which can help protect against certain types of bacteria that cause pneumonia. Practice good oral hygiene by brushing your teeth twice a day and flossing daily. Avoid smoking and secondhand smoke. Maintain a healthy lifestyle by eating a balanced diet and getting regular exercise. When to Call the Doctor If your symptoms worsen or you experience any of the following, call your doctor right away: Difficulty breathing High fever (over 102°F) Confusion Bluish tint to your lips or fingernails Additional Resources For more information on Pneumonia, you can visit these websites from reputable organizations: Centers for Disease Control and Prevention (CDC): https://www.cdc.gov/pneumonia/index.html National Heart, Lung, and Blood Institute: https://www.nhlbi.nih.gov/health/pneumonia Remember: This information is intended for general knowledge only and does not substitute for professional medical advice. Always consult with your doctor for diagnosis and treatment.

**Table 4:** Patient education guides generated for “pharyngitis”

Write a patient education guide for “Pharyngitis”	
ChatGPT	Google Gemini
Introduction: Pharyngitis, commonly known as a sore throat, is the inflammation of the throat, typically caused by viral or bacterial infections. This guide aims to provide you with essential information on understanding, managing, and preventing pharyngitis. Understanding Pharyngitis: Pharyngitis can be caused by various factors, including viruses (such as the common cold or flu) and bacteria (such as Streptococcus pyogenes). It is a common condition characterized by symptoms like sore throat, difficulty swallowing, fever, swollen glands in the neck, and sometimes coughing or sneezing. Symptoms of Pharyngitis: Sore throat: Pain or discomfort in the throat, especially when swallowing. Difficulty swallowing: Feeling of obstruction or pain while swallowing food or liquids. Fever: Elevated body temperature, often accompanied by chills or sweating. Swollen glands: Enlargement of lymph nodes in the neck, resulting in tenderness or soreness. Coughing or sneezing: Some individuals may experience coughing or sneezing, particularly if the pharyngitis is associated with a respiratory infection. Management and Treatment: Rest: Get plenty of rest to allow your body to recover from the infection. Hydration: Drink plenty of fluids, such as water, herbal tea, or broths, to stay hydrated and soothe the throat. Gargling: Gargle with warm salt water several times a day to reduce throat inflammation and relieve discomfort. Pain relief: Over-the-counter pain relievers like acetaminophen (Tylenol) or ibuprofen (Advil, Motrin) can help alleviate sore throat pain and reduce fever. Throat lozenges or sprays: Use throat lozenges or sprays containing ingredients like menthol or benzocaine to temporarily numb the throat and provide relief. Avoid irritants: Avoid smoking, secondhand smoke, and other irritants that can aggravate the throat and prolong recovery. When to Seek Medical Attention: Persistent symptoms: If symptoms persist for more than a few days or worsen over time. Difficulty breathing or swallowing: Seek immediate medical attention if you experience difficulty breathing or swallowing. Recurrent infections: If you experience frequent episodes of pharyngitis, your healthcare provider may recommend further evaluation to identify underlying causes. Complications: Rare complications of pharyngitis include rheumatic fever, peritonsillar abscess, or scarlet fever, which may require medical intervention. Preventive Measures: Good hygiene: Wash your hands frequently, especially before eating or touching your face, to prevent the spread of infections. Avoid close contact: Avoid close contact with individuals who have respiratory infections, and practice respiratory hygiene, such as covering your mouth and nose when coughing or sneezing. Stay healthy: Maintain a healthy lifestyle with a balanced diet, regular exercise, and adequate sleep to support your immune system and reduce the risk of infections. Conclusion: Pharyngitis is a common condition that can cause discomfort and inconvenience, but with proper self-care and preventive measures, most cases can be managed effectively at home. However, it's important to seek medical attention if symptoms are severe, persistent, or associated with difficulty breathing or swallowing. By taking proactive steps to manage and prevent pharyngitis, you can minimize its impact on your health and well-being.	Understanding Pharyngitis (Sore Throat) Pharyngitis, commonly known as a sore throat, is an irritation and inflammation of the pharynx, the part of your throat that connects your nose and mouth to your windpipe. It's a very common condition, especially during cold and flu season. This guide will explain pharyngitis, including its causes, symptoms, treatment options, and how to prevent it. What Causes Pharyngitis? There are two main causes of pharyngitis: Viruses: Viral infections are the most common cause, often associated with the common cold or flu. Bacteria: Group A Streptococcus (strep throat) is the most common bacterial cause. What are the Symptoms? Symptoms of pharyngitis can vary depending on the cause. Here are some common ones: Scratchy or sore throat Pain that worsens when swallowing Difficulty swallowing Swollen lymph nodes in the neck Fever (more common with bacterial infections) Cough (more common with viral infections) How is Pharyngitis Diagnosed? Your doctor can usually diagnose pharyngitis with a physical exam and by asking about your symptoms. In some cases, they might do a rapid strep test to quickly identify a bacterial infection. How is Pharyngitis Treated? There is no specific cure for pharyngitis, but treatments can help manage symptoms and speed up recovery. Here's what to expect: Viral Pharyngitis: Since antibiotics won't help viruses, treatment focuses on relieving symptoms. This may include over-the-counter pain relievers like acetaminophen or ibuprofen, lozenges, gargling with warm salt water, and using a cool mist humidifier. Bacterial Pharyngitis (Strep Throat): Your doctor will prescribe antibiotics to clear the infection and prevent complications. It's crucial to finish the entire course of antibiotics, even if you start feeling better. Preventing Pharyngitis Frequent handwashing: This is the best way to prevent the spread of germs that cause pharyngitis. Avoid secondhand smoke: Smoke can irritate your throat. Get enough rest: This allows your body to focus on healing. Stay hydrated: Drinking plenty of fluids helps soothe a sore throat. When to See a Doctor See your doctor if: Your sore throat is severe and doesn't improve within a few days. You have a fever over 101°F (38.4°C). You have difficulty swallowing. You have swollen lymph nodes that last for more than two weeks. You have a severe earache or headache. Remember: This guide is for informational purposes only. Always consult with your doctor for diagnosis and treatment recommendations.
